# Microarray data on altered transcriptional program of *Phgdh*-deficient mouse embryonic fibroblasts caused by ʟ-serine depletion

**DOI:** 10.1016/j.dib.2016.04.052

**Published:** 2016-04-26

**Authors:** Momoko Hamano, Tomoko Sayano, Wataru Kusada, Hisanori Kato, Shigeki Furuya

**Affiliations:** a Laboratory of Functional Genomics and Metabolism, Departments of Innovative Science and Technology for Bio-industry, Graduate School of Bioresource and Bioenvironmental Sciences, Kyushu University, Fukuoka 812-8581, Japan; b Innovative Bio-Architecture Center, Kyushu University, Fukuoka 812-8581, Japan; c International College of Arts and Sciences, Fukuoka Women׳s University, Fukuoka 813-8529, Japan; d Laboratory for Molecular Membrane Neuroscience, RIKEN Brain Science Institute, Wako, Saitama 351-0198, Japan; e Corporate Sponsored Research Program “Food for Life”, Organization for Interdisciplinary Research Projects, The University of Tokyo, Tokyo 113-8657, Japan

**Keywords:** Serine, PHGDH, Microarray, Inborn error, Neu-Laxova syndrome

## Abstract

Inherent ʟ-Ser deficiency culminates in intrauterine growth retardation, severe malformation of multiple organs particularly the central nervous system, and perinatal or early postnatal death in human and mouse. To uncover the molecular mechanisms underlying the growth-arrested phenotypes of l-Ser deficiency, we compared gene expression profiles of mouse embryonic fibroblasts deficient in 3-phosphoglycerate dehydrogenase (*Phgdh*), the first enzyme of de novo ʟ-Ser synthetic pathway, between ʟ-Ser-depleted and -supplemented conditions. The datasets (CEL and CHP files) from this study are publicly available on the Gene Expression Omnibus repository (accession number GEO: GSE55687).

**Specifications Table**

**Table:** 

Subject area	Biology
More specific subject area	Molecular Biology, Nutritional Biochemistry
Type of data	Table, Figure
How data was acquired	Microarray data generated on Affymetrix Mouse Genome 430 2.0 GeneChip Array
Data format	Analyzed
Experimental factors	Comparison of gene expression profiles of *Phgdh*-deficient embryonic fibroblasts between ʟ-Ser-supplemented and -depleted conditions
Experimental features	RNA isolation, global gene expression analysis, and bioinformatics analyses using IPA and DAVID
Data source location	Laurel, MD, USA
Data accessibility	Dataset is within this article and available in the Gene Expression Omnibus with accession number GEO: GSE55687.

**Value of the data**

• The gene expression data list the significantly affected genes by reduced ʟ-Ser availability in *Phgdh*-deficient mouse embryonic fibroblasts.
• Enriched GO terms and phenotypically relevant gene networks provide insight into altered cholesterol metabolism and stress responses elicited by l-Ser deficiency in embryonic fibroblasts.
• The data suggest that *Phgdh*-deficient mouse embryonic fibroblasts serve as a valuable mouse cellular model for human inborn ʟ-Ser deficiency including Neu-Laxova syndrome.

## 1 Data

**Fig. 1:**
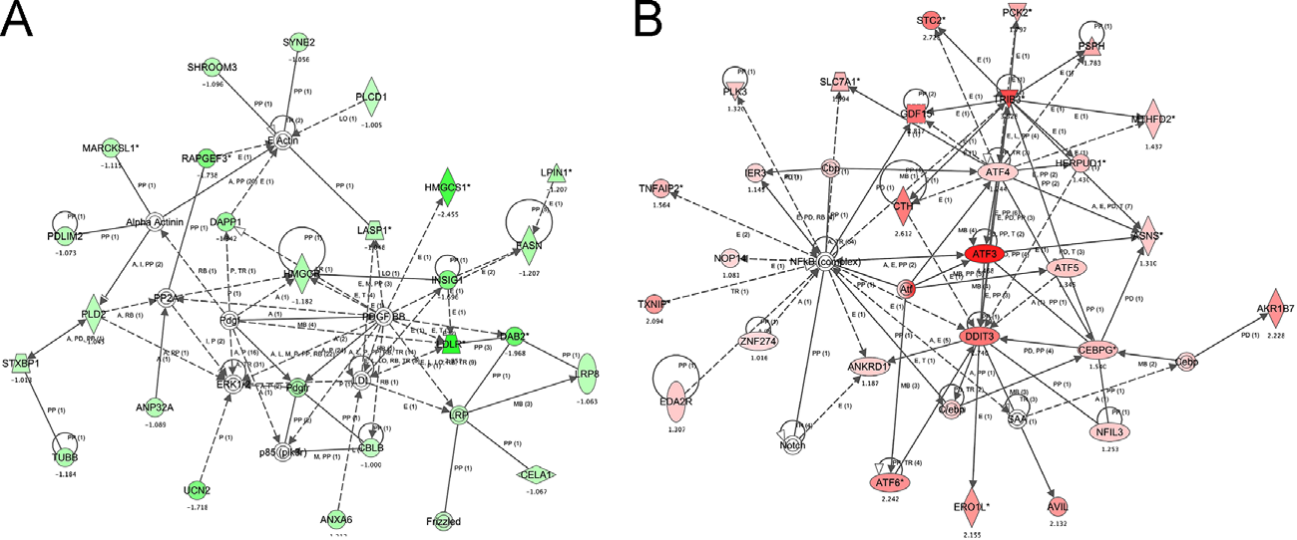
Phenotypically relevant gene networks enriched in *Phgdh*-deficient MEFs under ʟ-Ser-depleted condition. Gene lists were analyzed by the Ingenuity Pathway Analysis software to identify the top phenotypically relevant gene networks in down-regulated genes (A) and up-regulated genes (B). The networks are displayed graphically as nodes (genes/proteins) and edges (biological interactions between the nodes). The node color intensity indicates the degree of down- (green) or up- (red) regulation. Nodes are displayed using various shapes representing the functional class of the gene product. Edges are displayed with various labels that present the biological nature of interactions between the nodes as follows: A, activation; B, binding; E, expression; I, inhibition; LO, localization; P, phosphorylation/dephosphorylation; PD, protein–DNA binding; PR, protein–mRNA binding; PP, protein–protein binding; T, transcription. Straight lines indicate direct interactions, and dashed lines indicate indirect interactions. Edges without a label represent binding only.

Table 1 of DAVID analysis shows that cholesterol/sterol biosynthetic/metabolic process was enriched GO terms in the biological process (BP) of the down-regulated 381 genes, whereas apoptosis/cell death, amino acid biosynthetic process, tRNA aminoacylation, cell cycle arrest, and transcription were major significantly enriched GO terms in the up-regulated 560 genes. Ingenuity Pathway Analysis (IPA) determined top-ranked networks in the down-regulated genes (Fig. 1A) and the up-regulated genes (Fig. 1B). A network containing genes involved in the cholesterol metabolic process including *Hmgcs1*, *Insig*, *Hmgcr, and Ldlr,* was markedly diminished in the down-regulated genes, while the activation of a network containing stress-responsive *Atf4*–*Atf3*–*Ddit3* (CHOP) axis was most prominently in the up-regulated genes.

## 2 Experimental design, materials and methods

### 2.1 Cells

*Phgdh*-deficient MEFs were established from individual E13.5 embryos of *Phgdh* KO mice and maintained as described [1], [2]. To deplete ʟ-Ser, the complete DMEM medium was replaced with Eagle׳s Minimum Essential medium lacking ʟ-Ser and other non-essential amino acids with Earle׳s salts (EMEM; Wako Pure Chemical Industries Ltd.) supplemented with 1% FBS and 10 µg/ml gentamicin [1], [2]. When supplemented ʟ-Ser, 400 µM ʟ-Ser was added to this 1% FBS–EMEM medium.

### 2.2 Microarray analysis

Total RNA was extracted using the RiboPure kit (Thermo Fisher Scientific, Waltham, MA USA) after a 6 h incubation under ʟ-Ser-depleted or -supplemented conditions as described [1]. cDNA amplification and labeling, and chip hybridization were carried out as described [1]. After washing, the arrays were scanned with a GeneChipScanner (Affymetrix), and the scans data were processed using the GeneSuite software (Affymetrix). Three biological replicates for each treatment were directly compared.

### 2.3 Data processing and statistical analysis

The data in .CEL files were transferred to GeneSpring 8.0 software (Agilent Technologies). After normalization to its median value, filtration was performed based on the following criteria: (i) scaled intensity>100 under at least one condition; (ii) false discovery rate, *q*<0.01; and (iii) absolute value of fold change (ʟ-Ser-depleted condition/ʟ-Ser-supplemented) >2.0 or <0.5.

### 2.4 GO term enrichment and pathway analysis

Significantly differentially expressed genes were analyzed using the Database for Annotation, Visualization, and Integrated Discovery (DAVID) to calculate GO term enrichments in the biological process category at all levels [3]. Enriched GO terms (Benjamini–Hochberg correction:*Q*-value<0.05) were deemed significant. Phenotypically relevant gene networks of significantly differentially expressed genes were analyzed using the web-based expression analysis program Ingenuity Pathways Analysis (http://www.ingenuity.com).

## Figures and Tables

**Table 1:** Enriched GO terms in mRNA transcripts of *Phgdh*-deficient MEFs elicited by ʟ-Ser depletion.

	Term	Count	%	*P*-value	*Q*-value

**Down**	**GO:0006695 Cholesterol biosynthetic process**	**6**	**2.0**	**1.46E–05**	**0.018**
**GO:0008203 Cholesterol metabolic process**	**8**	**2.6**	**5.70E–05**	**0.035**
**GO:0016126 Sterol biosynthetic process**	**6**	**2.0**	**5.72E–05**	**0.024**
**GO:0016125 Sterol metabolic process**	**8**	**2.6**	**1.05E–04**	**0.033**
**Up**	**GO:0042981 Regulation of apoptosis**	**33**	**8.5**	**3.21E–08**	**5.30E–05**
**GO:0043067 Regulation of programmed cell death**	**33**	**8.5**	**4.29E–08**	**3.55E–05**
**GO:0010941 Regulation of cell death**	**33**	**8.5**	**4.89E–08**	**2.70E–05**
**GO:0006916 Anti-apoptosis**	**11**	**2.8**	**7.70E–06**	**0.003**
**GO:0008652 Cellular amino acid biosynthetic process**	**8**	**2.1**	**2.00E–05**	**0.007**
**GO:0044271 Nitrogen compound biosynthetic process**	**19**	**4.9**	**2.56E–05**	**0.007**
**GO:0043038 Amino acid activation**	**8**	**2.1**	**2.70E–05**	**0.006**
**GO:0006418 tRNA aminoacylation for protein translation**	**8**	**2.1**	**2.70E–05**	**0.006**
**GO:0043039 tRNA aminoacylation**	**8**	**2.1**	**2.70E–05**	**0.006**
**GO:0043066 Negative regulation of apoptosis**	**16**	**4.1**	**6.84E–05**	**0.014**
**GO:0006399 tRNA metabolic process**	**11**	**2.8**	**6.92E–05**	**0.013**
**GO:0043069 Negative regulation of programmed cell death**	**16**	**4.1**	**8.65E–05**	**0.014**
**GO:0060548 Negative regulation of cell death**	**16**	**4.1**	**9.00E–05**	**0.013**
**GO:0010557 Positive regulation of macromolecule biosynthetic process**	**25**	**6.4**	**1.06E–04**	**0.015**
**GO:0007050 Cell cycle arrest**	**8**	**2.1**	**1.11E–04**	**0.014**
**GO:0034976 Response to endoplasmic reticulum stress**	**6**	**1.5**	**1.25E–04**	**0.015**
**GO:0012501 Programmed cell death**	**23**	**5.9**	**1.42E–04**	**0.015**
**GO:0008219 Cell death**	**24**	**6.2**	**1.45E–04**	**0.015**
**GO:0006357 Regulation of transcription from RNA polymerase II promoter**	**27**	**6.9**	**1.70E–04**	**0.016**
**GO:0031328 Positive regulation of cellular biosynthetic process**	**25**	**6.4**	**1.96E–04**	**0.018**
**GO:0045449 Regulation of transcription**	**67**	**17.2**	**1.97E–04**	**0.017**
**GO:0016265 Death**	**24**	**6.2**	**2.03E–04**	**0.017**
**GO:0009891 Positive regulation of biosynthetic process**	**25**	**6.4**	**2.25E–04**	**0.018**
**GO:0030968 Endoplasmic reticulum unfolded protein response**	**5**	**1.3**	**2.72E–04**	**0.020**
**GO:0034620 Cellular response to unfolded protein**	**5**	**1.3**	**2.72E–04**	**0.020**
**GO:0006915 Apoptosis**	**22**	**5.7**	**2.99E–04**	**0.021**
**GO:0006355 Regulation of transcription, DNA-dependent**	**48**	**12.3**	**3.68E–04**	**0.025**
**GO:0045935 Positive regulation of nucleobase, nucleoside, nucleotide and nucleic acid metabolic process**	**23**	**5.9**	**4.06E–04**	**0.026**
**GO:0051252 Regulation of RNA metabolic process**	**48**	**12.3**	**5.20E–04**	**0.033**
**GO:0009309 Amine biosynthetic process**	**8**	**2.1**	**5.27E–04**	**0.032**
**GO:0051173 Positive regulation of nitrogen compound metabolic process**	**23**	**5.9**	**6.16E–04**	**0.036**
